# Esophagus cancer and essential trace elements

**DOI:** 10.3389/fpubh.2022.1038153

**Published:** 2022-11-16

**Authors:** Xin Yang, Zhe Tang, Jing Li, Jizong Jiang

**Affiliations:** ^1^Department of Oncology, Tongji Hospital of Tongji Medical College, Huazhong University of Science and Technology, Wuhan, China; ^2^Department of Thoracic Surgery, Tongji Hospital of Tongji Medical College, Huazhong University of Science and Technology, Wuhan, China

**Keywords:** esophagus cancer, essential trace elements, minerals, zinc, copper, iron, selenium

## Abstract

Numerous epidemiological and laboratory studies on essential trace elements have reported protective associations in developing various cancer types, including esophagus cancer (EC). However, the results are not always consistent. Some essential trace elements could play a vital role in preventing esophagus cancer. Some showed no association with esophageal cancer risk, while others harmed individuals. This article reviews the association between the intake or supplementation of essential trace elements (especially zinc, copper, iron, and selenium) and the risk of esophageal cancer. Generally, zinc intake may decrease the risk of esophageal cancer (EC), especially in high esophageal squamous cell carcinoma (ESCC) prevalence regions. The association between copper supplementation and EC remains uncertain. Total iron consumption is thought to be associated with lower EC risk, while heme iron intake may be associated with higher EC risk. Selenium intake showed a protective effect against EC, especially for those individuals with a low baseline selenium level. This review also prospects the research direction of the association between EC and essential trace elements.

## Introduction

Esophageal cancer (EC) is the seventh leading cause of cancer death worldwide (1) and the fourth leading cause of cancer death in China (2). The 5-year survival rate for EC remains one of the lowest in all cancer types, with a 5-year survival of 20% in the United States (1) and 40.1% in China (3). More than 90% of EC patients in China are diagnosed with esophageal squamous cell carcinoma (ESCC) (3, 4), the world's most common type of EC. In Western nations, alcohol drinking and smoking are the primary risk factors for ESCC (5), whereas, in so-called “esophageal cancer belts” such as South Africa, France, Iran, and China, these behaviors are less prevalent (6). Environmental and dietary factors have also been reported to affect ESCC, and essential trace elements may be dose-dependent on the risk of ESCC. Essential trace elements of the human body account for roughly 0.00001% to 0.01% of the total body weight, including Fe, Zn, Cu, Mo, F, V, Ni, Co, Se, Cr, I, and Mn. They play a significant role in maintaining normal biological function, acting as active centers of enzymes or tracing bioactive substances. Numerous studies have attempted to illustrate the meaning of balancing essential trace elements in EC protection or treatment. Yet, the findings of these studies are not always the same. Here, we focus on the connection of esophagus cancer with essential trace elements, especially zinc, copper, iron, and selenium.

## Zinc

Zinc is one of the most abundant trace elements found in almost all organs and tissues of the body. It plays a critical role in stabilizing the structures of many proteins, especially those participating in DNA synthesis and RNA transcription, thus regulating cell growth, development, and differentiation, maintaining an immune response, and mediating oxidative stress and apoptosis (7–9). Among all the biological functions, it should be highlighted that zinc could inhibit chronic or acute oxidative stress, which is one of the mechanisms of cancer development. Zinc deficiency impairs the antioxidant activity and renders the organism more susceptible to injury induced by various oxidative stressors (10). Therefore, an adequate level of zinc is important for individuals. Some laboratory studies have established an association between zinc deficiency and cancer (11–16). Zinc homeostasis could influence T-cell activation as well as the polarization of T helper (Th) cells into their different subpopulations [Th1, Th2, Th17, regulatory T cells (Treg)], thus regulating cancer immune microenviroment (15). Choi et al. found that zinc could inhibit cell proliferation of EC cells through Orai1-mediated intracellular Ca^2+^ oscillations and revealed a possible molecular basis for zinc-induced cancer prevention and the Orai1-SOCE signaling pathway in cancer cells (16). Several studies have already shown the relationship between dietary zinc deficiency and ESCC (17, 18). ESCC in Linxian is a high-incidence area in China. A study from Linxian showed that the zinc concentration in biopsy samples was negatively correlated with the venture of EC development, exhibiting strong proof for the connection of dietary zinc deficiency with the higher risk of EC in humans (17).

Zinc consumption was significantly associated with a lower risk of EC and gastric cancer in Asia but not in the United States or Europe (19). The results of a meta-analysis also indicated that increasing zinc supplementation by 5 mg/day was associated with a 15% decrease in EC risk (20). It was reported that the mechanisms between zinc deficiency and the development of EC lie in that zinc deficiency could result in the upregulation of multiple genes related to DNA damage, oxidative stress, immune response, cell proliferation, and apoptosis, thus inducing the development of EC (18). Animal studies showed that ESCC cells proliferated more rapidly in mice fed zinc-deficient diets *via* inducing overexpression of COX-2, P38, PCNA, and NF-κB (18). ESCC can also be promoted by zinc deficiency through inflammatory gene expression (21, 22) and oncogenic microRNA expression, including upregulation of oncogenic miR-31 and other miRNAs (21, 23). Deeper molecular mechanisms induced by nutrient bioavailability or dietary interventions have recently been studied using integrative genomics methods (24). Fong et al. (25) demonstrated that zinc intake reduced COX-2 mRNA by 80%, an enzyme involved in inflammation, thus bringing prevention or therapeutic possibilities of zinc supplementation for EC. A recent study found that zinc supplementation could protect Barrett's epithelia from transforming into esophageal cancer cells (26), further revealing zinc supplementation's protective effects. The role of zinc in EC diseases is not fully answered and needs further investigation. It seems that zinc intake may decrease EC risk, especially in high ESCC prevalence regions.

## Copper

Copper is an essential trace element that the body requires and is vital in many biological functions, including maintaining DNA integrity, synthesizing essential metabolites, transporting oxygen to the mitochondrial respiratory chain, and involving redox reactions as an active site metabolic cofactor. Emerging laboratory studies showed that copper could act as a dynamic signaling metal and metalloallosteric regulator, participating in cell growth and proliferation, autophagy, and antioxidant defense, thus regulating cancer development, as tumor growth and metastasis have a high requirement for this metal nutrient (27). Such properties make the dual copper effect beneficial and toxic to the cells. An intake of 900 μg per day is recommended for this essential trace element, while a level of 10 mg per day is the maximum permissible (28). Excess copper may promote radical damage and decrease the activity of proteins or enzymes, thus causing cellular injury *via* over-activated oxidative stress, lipid peroxidation, inflammation, and DNA damage, finally helping in the angiogenesis of tumors (29). It remains controversial whether copper intake contributes to EC prevention, despite many studies focusing on the relationship between copper intake and the disease. Chen et al. (30) claimed that copper intake was negatively correlated with EC mortality in Shanxi, China, *via* estimating average copper intake in 21 Chinese communes, where EC mortality rates were much higher than average (30). Similarly, according to Sohrabi et al. (31) the copper levels were obviously lowered in EC tissues compared to non-cancerous tissues (31). Recently, Zhuang et al. (32) even explored the therapeutic effect of copper nanoparticles in EC treatment *via* their antioxidant activities (32). However, in the Kashmir valley, an area at high risk for EC in India, as reported by Mir et al. (33) plasma levels of copper in patients with EC were significantly higher than in controls, indicating an imbalance in plasma levels of copper may be responsible for the development of the disease (33). Copper supplementation and ESCC risk were evaluated by Hashemian et al. (34) and a nonlinear association was found between copper intake and ESCC risk, but the supplementation trend related to ESCC was not evident (34). In addition, a meta-analysis found that copper supplementation at 1 mg/day did not reduce the risk of EC (20). Altogether, the relationship between copper and EC remains uncertain. Copper supplementation recommendations still require additional research.

## Iron

Iron is an important component of heme, iron-sulfur proteins, and enzymes and participates in many biological processes, including oxygen transport, ferroptosis, immune response, cellular energy metabolism, and many other enzymatic reactions. Physiological processes have evolved for iron acquisition to meet metabolic needs while avoiding the toxicity of free radicals generated from iron. Iron excess and iron deficiency are related to pathological states (35). Recent studies have focused on ferroptosis, a newly discovered iron-dependent mode of cell death that plays an important role in the biological behavior of cancer cells. Zhu et al. (36) and Qiao et al. (37) found that ferroptosis-related noncoding RNAs correlate with the prognosis, tumor microenvironment, and therapeutic sensitivity of ESCC, indicating new therapeutic approaches for noncoding RNAs targeting ferroptosis in ESCC (36, 37). The role of iron in EC development has been under investigation. However, the results are still conflicting regarding the association of iron levels with the risk of EC. Sohrabi et al. (31) evaluated the iron concentrations between cancerous and non-cancerous tissues in EC. The results showed that the iron level in cancerous tissues was higher. However, no significant differences were revealed in EC (31). Several basic research indicated that the excess levels of iron enhanced EC (38–40), and the mechanisms possibly were related to the over-expression of iron import proteins, DNA damage, ferroptosis, and oxidative stress (41, 42).

Furthermore, it was found that total iron intake and heme iron intake were different from the risk of EC. Total iron supplementation was significantly inversely correlated with the risk of EC, especially among the Asian population and ESCC subgroup. A dose-response analysis indicated that each 5 mg/day increase in total iron supplementation was related to a 15% reduction in EC risk. However, heme iron intake was positively related to the risk of EC, especially in the United States. Each 1 mg/day increase in heme iron supplementation increased the risk of EC by 21% (20).

Similarly, a large European cohort study found that higher intakes of processed meat and heme iron may be related to an increased risk of developing EC, especially esophageal adenocarcinoma (43). Cross et al. (42) also found that heme iron supplementation may be associated with a risk for esophageal adenocarcinoma (EAC). They observed a positive association between red meat intake and ESCC but no association with adenocarcinoma (42). Overall, total iron intake may be related to reduced EC risk, while heme iron intake may be related to increased EC risk. More evidence is required to clarify the relationship between imbalanced iron levels (iron deficiency or overload) and the risk of EC.

## Selenium

Selenium is a naturally occurring element to which humans are mostly exposed through food intake, air, drinking water, and dietary supplements. A moderate amount of selenium is vital for maintaining biological functions, but a slightly higher amount of selenium may have potential toxicity. Basic research showed an anti-tumor effect of selenium, including inhibiting cancer cell proliferation (44), preventing tumor formation in cell populations already exposed to carcinogens (45), reducing carcinogen-induced DNA mutations, and antioxidant and anti-inflammatory effects (46).

Numerous studies have shown selenium to be related to the risk of EC. It was reported that selenium could slow down the development of ESCC by decreasing the expression of Ki-67, inducing apoptosis, and lowering inflammation and oxidative DNA damage, thus exerting an important chemopreventive effect on ESCC by reducing high-grade dysplasia to low-grade dysplasia (47, 48). Zhang et al. (49) found that β-catenin/TCF pathway played a vital role in selenium induced-growth inhibition and apoptosis in ESCC cells (49). Liu et al. (50) also revealed that methylseleninic acid acted as a chemopreventive agent *via* the regulation of KLF4/miR-200a/Keap1/Nrf2 axis in ESCC cells (50). Several studies measured serum selenium levels between patients with EC and controls. Steevens et al. detected selenium levels in toenails. They found that selenium concentrations were inversely related to the risk of ESCC, while an inverse association was only found in esophageal adenocarcinoma (EAC) in women and non-smokers (51). Mark et al. (52) found a significant inverse relationship between serum selenium concentrations and the incidence of EC (52).

Further, they also observed significant inverse associations between the baseline concentration of serum selenium and death from ESCC (53), indicating that selenium intake may help reduce the incidence of EC and death from EC. A randomized controlled trial showed evidence that selenium played a preventive role in subjects with preexisting esophageal squamous dysplasia, which was reported as the precursor lesion of ESCC (54). In a follow-up of the Linxian General Population Nutrition Intervention Trial, selenium intake with vitamin E and β-carotene helped reduce the risk of EC, and the beneficial effects on mortality from EC were still evident up to 10 years after the cessation of supplementation and were consistently greater in younger patients (55). However, some meta-analyses showed different results regarding the relationship between selenium exposure and the risk of EC. Cai et al. (56) showed that high selenium exposure might decrease the risk of EC (56), while Hong et al. (57) observed that a higher selenium concentration was not significantly related to a decreased risk of EC (57). The Golestan Cohort Study also mentioned that the association between dietary selenium supplementation and the risk of ESCC was nonlinear but probably U-shaped, which suggests that the risk of ESCC may increase with excessive selenium intakes (34). Generally, for those individuals with a low baseline selenium level, selenium intake could have a protective effect against EC; for general populations, the effect of daily supplementation of selenium remains unclear.

## Other essential trace elements

In a cohort study, calcium (Ca) was reported to be related to a decreased risk of ESCC in men in the United States (58). Similarly, Hashemian et al. (34) observed a significant linear inverse association between calcium supplementation and the risk of ESCC (34). The possible molecular mechanisms may lie in the fact that calcium could inhibit the proliferation and invasion of cancer cells and promote apoptosis (59). The relationship between magnesium (Mg) in drinking water and the risk of EC was also reported. The result showed a significant trend toward decreasing EC risk with increasing magnesium concentration in drinking water (60). It was also reported that compared with healthy tissues, the levels of chromium (Cr), manganese (Mn), aluminum (Al), tin (Sn), and lead (Pb) were higher in cancerous tissues. However, no significant differences were revealed in EC (31). There are only limited studies focused on the relationship between the aforementioned essential trace elements and the risk of EC. Cr was reported to increase the risk of various cancer infections under environmental and occupational exposures (61) *via* ROS production, DNA damage, angiogenesis, and other molecular process (62, 63); however, its association with EC remains unclear. Mn has been widely studied in neurodegenerative diseases, and it mainly participates in biological roles in the form of manganese superoxide dismutase (MnSOD) by neutralizing the radical superoxide. A few studies have reported that MnSOD may play a role in EC protection (64, 65). In a mouse model, high levels of MnSOD expression promoted ESCC cell growth, whereas moderate MnSOD expression suppressed tumor cell growth (64), indicating the dual effects of MnSOD on ESCC cell proliferation. No evidence showed a clear relationship between Al, Sn, Pb, and EC. Further study may reveal the underlying association.

## Discussion

**Figure 1:**
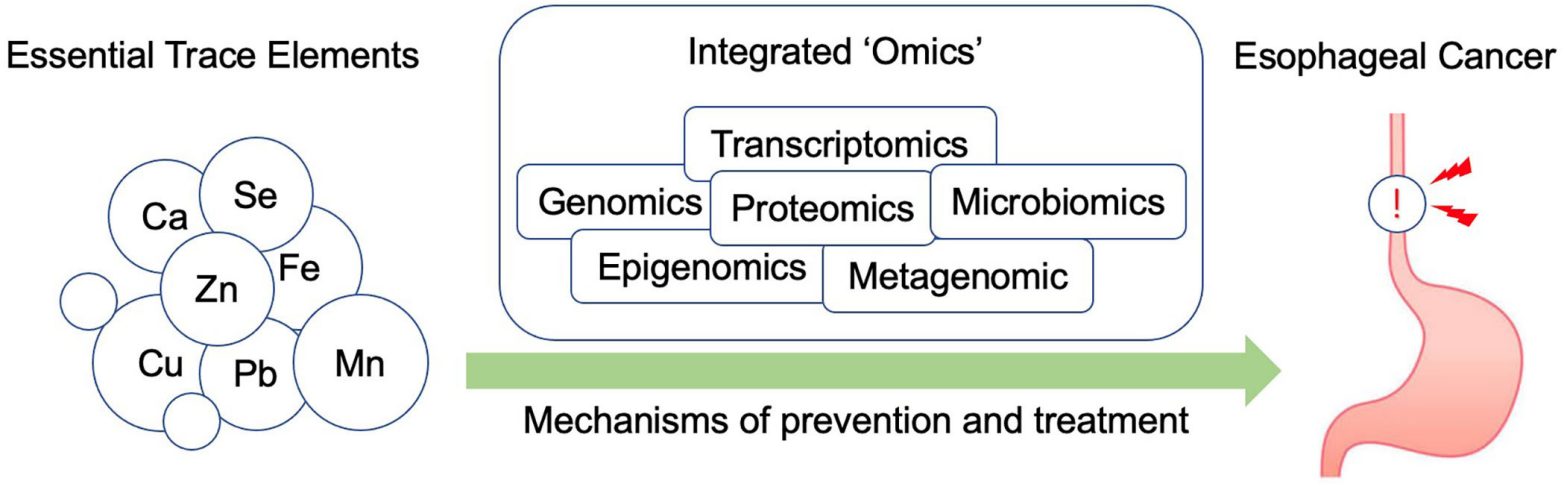
An overview of how integrated omics technologies could be applied to study the relationship between essential trace elements and EC.

It was found that EC had a close relationship with environmental and dietary factors, in which essential trace elements played significant roles. Studies on the association between essential trace elements and EC were critical and have provided potential direction for the prevention of EC. In this review, we analyzed the studies of some main essential trace elements in EC. Zinc, iron, and selenium supplementation seem to be related to a reduced risk of EC, while copper showed an equivocal effect on the prevention of EC. Despite ample evidence and general consistency in the relationship between essential trace elements and EC, the public or health professionals often view the effect of essential trace elements with skepticism. One explanation could be that the physiologic systems affected by essential trace elements are so complicated that the effects of supplementing with only one or two elements are not effective enough or even sometimes harmful. Essential trace elements belong to the whole diet pattern. Many studies focused on only one essential trace element, which could not reflect the reality that diet as a whole is more important than the sum of its parts (66). In-depth studies on essential trace elements are required. Genome, epigenome, transcriptome, metabolome, proteome, and microbiome have long been used in cancer studies, which significantly improve our understanding of the mechanisms of cancer development or new therapy application of cancers. With the development of such technologies, a molecular biological approach that integrates data of “omics,” including metagenomics, transcriptomics, proteomics, metabolomics, genetics, and other molecular technology, is expected to help us enhance our understanding of the association between essential trace elements and risk of EC incidence or development (66) (Figure 1). Moreover, a clearer connection between essential trace elements and the molecular alteration and variation of patients is gradually becoming identifiable and quantifiable, thereby renewing the old general view associating specific phenotypical changes with the differential intake of essential trace elements (67). Further research is needed and is expected to clarify how essential trace elements act in the development of EC and whether essential trace element supplementation could protect against EC.

## Author contributions

XY carried out the primary literature search, drafted, and revised the manuscript. JL and ZT contributed to drafting and revising of the manuscript. JJ and JL helped modify the manuscript. ZT and JJ carried out the literature analysis and revised the manuscript. All authors read and approved the final manuscript.

## Conflict of interest

The authors declare that the research was conducted in the absence of any commercial or financial relationships that could be construed as a potential conflict of interest.

## Publisher's note

All claims expressed in this article are solely those of the authors and do not necessarily represent those of their affiliated organizations, or those of the publisher, the editors and the reviewers. Any product that may be evaluated in this article, or claim that may be made by its manufacturer, is not guaranteed or endorsed by the publisher.

## References

[1] Siegel RL Miller KD Fuchs HE Jemal A. Cancer statistics, 2022. CA Cancer J Clin. (2022) 72:7–33. 10.3322/caac.21708 35020204

[2] Jiang D Zhang L Liu W Ding Y Yin J Ren R. Trends in cancer mortality in China from 2004 to 2018: A nationwide longitudinal study. Cancer Commun. (2021) 41:1024–36. 10.1002/cac2.12195 34251754 PMC8504142

[3] Hou H Meng Z Zhao X Ding G Sun M Wang W. Survival of esophageal cancer in China: a pooled analysis on hospital-based studies from 2000 to 2018. Front Oncol. (2019) 9:548. 10.3389/fonc.2019.00548 31316913 PMC6610307

[4] Zhang X Peng L Luo Y Zhang S Pu Y Chen Y. Dissecting esophageal squamous-cell carcinoma ecosystem by single-cell transcriptomic analysis. Nat Commun. (2021) 12:5291. 10.1038/s41467-021-25539-x 34489433 PMC8421382

[5] Bravi F Edefonti V Randi G Garavello W La Vecchia C Ferraroni M. Dietary patterns and the risk of esophageal cancer. Ann Oncol. (2012) 23:765–70. 10.1093/annonc/mdr295 21653682

[6] Jessri M Rashidkhani B Hajizadeh B Jessri M Gotay C. Macronutrients, vitamins and minerals intake and risk of esophageal squamous cell carcinoma: a case-control study in Iran. Nutr J. (2011) 10:137. 10.1186/1475-2891-10-137 22185224 PMC3260093

[7] Chasapis CT Loutsidou AC Spiliopoulou CA Stefanidou ME. Zinc and human health: an update. Arch Toxicol. (2012) 86:521–34. 10.1007/s00204-011-0775-1 22071549

[8] Saper RB Rash R. Zinc: an essential micronutrient. Am Fam Physician. (2009) 79:768–72. 20141096 PMC2820120

[9] Schwartz MK. Role of trace elements in cancer. Cancer Res. (1975) 35:3481–7. 1104155

[10] Jarosz M Olbert M Wyszogrodzka G Mlyniec K Librowski T. Antioxidant and anti-inflammatory effects of zinc. Zinc Dep NF-kappaB Signal Inflammopharmacol. (2017) 25:11–24. 10.1007/s10787-017-0309-4 28083748 PMC5306179

[11] Federico A Iodice P Federico P Del Rio A Mellone MC Catalano G. Effects of selenium and zinc supplementation on nutritional status in patients with cancer of digestive tract. Eur J Clin Nutr. (2001) 55:293–7. 10.1038/sj.ejcn.1601157 11360134

[12] Prasad AS Beck FW Doerr TD Shamsa FH Penny HS Marks SC. Nutritional and zinc status of head and neck cancer patients: an interpretive review. J Am Coll Nutr. (1998) 17:409–18. 10.1080/07315724.1998.10718787 9791836

[13] Zowczak M Iskra M Torlinski L Cofta S. Analysis of serum copper and zinc concentrations in cancer patients. Biol Trace Elem Res. (2001) 82:1–8. 10.1385/BTER:82:1-3:001 11697759

[14] Prasad AS Beck FW Snell DC Kucuk O. Zinc in cancer prevention. Nutr Cancer. (2009) 61:879–87. 10.1080/01635580903285122 20155630

[15] Maywald M Rink L. Zinc homeostasis and immunosenescence. J Trace Elem Med Biol. (2015) 29:24–30. 10.1016/j.jtemb.2014.06.003 25022332

[16] Choi S Cui C Luo Y Kim SH Ko JK Huo X. Selective inhibitory effects of zinc on cell proliferation in esophageal squamous cell carcinoma through Orai1. FASEB J. (2018) 32:404–16. 10.1096/fj.201700227RRR 28928244 PMC6207365

[17] Abnet CC Lai B Qiao YL Vogt S Luo XM Taylor PR. Zinc concentration in esophageal biopsy specimens measured by x-ray fluorescence and esophageal cancer risk. J Natl Cancer Inst. (2005) 97:301–6. 10.1093/jnci/dji042 15713965

[18] Chen Y Liu FX Liu H. Effects of dietary zinc deficiency on esophageal squamous cell proliferation and the mechanisms involved. World J Gastrointest Oncol. (2021) 13:1755–65. 10.4251/wjgo.v13.i11.1755 34853648 PMC8603456

[19] Li P Xu J Shi Y Ye Y Chen K Yang J. Association between zinc intake and risk of digestive tract cancers: a systematic review and meta-analysis. Clin Nutr. (2014) 33:415–20. 10.1016/j.clnu.2013.10.001 24148607

[20] Ma J Li Q Fang X Chen L Qiang Y Wang J. Increased total iron and zinc intake and lower heme iron intake reduce the risk of esophageal cancer: A dose-response meta-analysis. Nutr Res. (2018) 59:16–28. 10.1016/j.nutres.2018.07.007 30442229

[21] Fong LY Farber JL Croce CM. Zinc intake, microRNA dysregulation, and esophageal cancer. Aging. (2016) 8:1161–2. 10.18632/aging.100978 27280381 PMC4931822

[22] Taccioli C Chen H Jiang Y Liu XP Huang K Smalley KJ. Dietary zinc deficiency fuels esophageal cancer development by inducing a distinct inflammatory signature. Oncogene. (2012) 31:4550–8. 10.1038/onc.2011.592 22179833 PMC3310953

[23] Liu CM Liang D Jin J Li DJ Zhang YC Gao ZY. Research progress on the relationship between zinc deficiency, related microRNAs, and esophageal carcinoma. Thorac Cancer. (2017) 8:549–57. 10.1111/1759-7714.12493 28892299 PMC5668500

[24] Kussmann M Raymond F Affolter M. OMICS-driven biomarker discovery in nutrition and health. J Biotechnol. (2006) 124:758–87. 10.1016/j.jbiotec.2006.02.014 16600411

[25] Fong LY Zhang L Jiang Y Farber JL. Dietary zinc modulation of COX-2 expression and lingual and esophageal carcinogenesis in rats. J Natl Cancer Inst. (2005) 97:40–50. 10.1093/jnci/dji006 15632379

[26] Valenzano MC Rybakovsky E Chen V Leroy K Lander J Richardson E. Zinc Gluconate induces potentially cancer chemopreventive activity in barrett's esophagus: a phase 1 pilot study. Dig Dis Sci. (2021) 66:1195–211. 10.1007/s10620-020-06319-x 32415564 PMC7677901

[27] Ge EJ Bush AI Casini A Cobine PA Cross JR DeNicola GM. Connecting copper and cancer: from transition metal signalling to metalloplasia. Nat Rev Cancer. (2022) 22:102–13. 10.1038/s41568-021-00417-2 34764459 PMC8810673

[28] Trumbo P Yates AA Schlicker S Poos M. Dietary reference intakes: vitamin A, vitamin K, arsenic, boron, chromium, copper, iodine, iron, manganese, molybdenum, nickel, silicon, vanadium, and zinc. J Am Diet Assoc. (2001) 101:294–301. 11269606 10.1016/S0002-8223(01)00078-5

[29] Nasulewicz A Mazur A Opolski A. Role of copper in tumour angiogenesis–clinical implications. J Trace Elem Med Biol. (2004) 18:1–8. 10.1016/j.jtemb.2004.02.004 15487757

[30] Chen F Cole P Mi Z Xing L. Dietary trace elements and esophageal cancer mortality in Shanxi, China. Epidemiology. (1992) 3:402–6. 10.1097/00001648-199209000-00004 1391131

[31] Sohrabi M Nikkhah M Sohrabi M Farimani AR Shahi MM Ziaie H. Evaluating tissue levels of the eight trace elements and heavy metals among esophagus and gastric cancer patients: a comparison between cancerous and non-cancerous tissues. J Trace Elem Med Biol. (2021) 68:126761. 10.1016/j.jtemb.2021.126761 34139544

[32] Zhuang X Kang Y Zhao L Guo S. Design and synthesis of copper nanoparticles for the treatment of human esophageal cancer: introducing a novel chemotherapeutic supplement. J Exp Nanosci. (2022) 17:274–84. 10.1080/17458080.2022.2065264

[33] Mir MM Dar NA Salam I Malik MA Lone MM Yatoo GN. Studies on association between copper excess, zinc deficiency and TP53 mutations in esophageal squamous cell carcinoma from Kashmir valley, India-a high risk area. Int J Health Sci. (2007) 1:35–42. 21475450 PMC3068648

[34] Hashemian M Poustchi H Abnet CC Boffetta P Dawsey SM Brennan PJ. Dietary intake of minerals and risk of esophageal squamous cell carcinoma: results from the golestan cohort study. Am J Clin Nutr. (2015) 102:102–8. 10.3945/ajcn.115.107847 26016858 PMC4480669

[35] Lal A. Iron in health and disease: an update. Indian J Pediatr. (2020) 87:58–65. 10.1007/s12098-019-03054-8 31520313

[36] Zhu J Zhao Y Wu G Zhang X Chen Q Yang B. Ferroptosis-related lncRNA signature correlates with the prognosis, tumor microenvironment, and therapeutic sensitivity of esophageal squamous cell carcinoma. Oxid Med Cell Longev. (2022) 2022:7465880. 10.1155/2022/7465880 35903713 PMC9315452

[37] Qiao G Zhang W Dong K. Regulation of ferroptosis by noncoding RNAs: a novel promise treatment in esophageal squamous cell carcinoma. Mol Cell Biochem. (2022) 477:2193–202. 10.1007/s11010-022-04441-0 35449482

[38] Chen X Yang G Ding WY Bondoc F Curtis SK Yang CS. An esophagogastroduodenal anastomosis model for esophageal adenocarcinogenesis in rats and enhancement by iron overload. Carcinogenesis. (1999) 20:1801–8. 10.1093/carcin/20.9.1801 10469627

[39] Nishitani S Noma K Ohara T Tomono Y Watanabe S Tazawa H. Iron depletion-induced downregulation of N-cadherin expression inhibits invasive malignant phenotypes in human esophageal cancer. Int J Oncol. (2016) 49:1351–9. 10.3892/ijo.2016.3640 27499208

[40] Shishido Y Amisaki M Matsumi Y Yakura H Nakayama Y Miyauchi W. Antitumor effect of 5-aminolevulinic acid through ferroptosis in esophageal squamous cell carcinoma. Ann Surg Oncol. (2021) 28:3996–4006. 10.1245/s10434-020-09334-4 33210267

[41] Chan KT Choi MY Lai KK Tan W Tung LN Lam HY. Overexpression of transferrin receptor CD71 and its tumorigenic properties in esophageal squamous cell carcinoma. Oncol Rep. (2014) 31:1296–304. 10.3892/or.2014.2981 24435655

[42] Cross AJ Freedman ND Ren J Ward MH Hollenbeck AR Schatzkin A. Meat consumption and risk of esophageal and gastric cancer in a large prospective study. Am J Gastroenterol. (2011) 106:432–42. 10.1038/ajg.2010.415 20978481 PMC3039705

[43] Jakszyn P Luján-Barroso L Agudo A. Bueno-de-Mesquita HB, Molina E, Sánchez MJ, et al. Meat and heme iron intake and esophageal adenocarcinoma in the European prospective investigation into cancer and nutrition study. Int J Cancer. (2013) 133:2744–50. 10.1002/ijc.28291 23728954

[44] Bertram JS Kolonel LN Meyskens FL. Rationale and strategies for chemoprevention of cancer in humans. Cancer Res. (1987) 47:3012–31. 3105872

[45] Steinmetz KA Potter JD. Vegetables, fruit, and cancer. II Mechanisms Cancer Causes Control. (1991) 2:427–42. 10.1007/BF00054304 1764568

[46] Rayman MP. Selenium and human health. Lancet. (2012) 379:1256–68. 10.1016/S0140-6736(11)61452-9 22381456

[47] Ahsan A Liu Z Su R Liu C Liao X Su M. Potential chemotherapeutic effect of selenium for improved canceration of esophageal cancer. Int J Mol Sci. (2022) 23:5509. 10.3390/ijms23105509 35628320 PMC9145868

[48] Liu T Sun Y Yang S Liang X. Inhibitory effect of selenium on esophagus cancer cells and the related mechanism. J Nutr Sci Vitaminol (Tokyo). (2020) 66:456–61. 10.3177/jnsv.66.456 33132349

[49] Zhang W Yan S Liu M Zhang G Yang S He S. Beta-Catenin/TCF pathway plays a vital role in selenium induced-growth inhibition and apoptosis in esophageal squamous cell carcinoma (ESCC) cells. Cancer Lett. (2010) 296:113–22. 10.1016/j.canlet.2010.04.001 20457486

[50] Liu M Hu C Xu Q Chen L Ma K Xu N. Methylseleninic acid activates Keap1/Nrf2 pathway via up-regulating miR-200a in human oesophageal squamous cell carcinoma cells. Biosci Rep. (2015) 35:5. 10.1042/BSR20150092 26341629 PMC4613709

[51] Steevens J Van den Brandt PA Goldbohm RA Schouten LJ. Selenium status and the risk of esophageal and gastric cancer subtypes: the Netherlands cohort study. Gastroenterology. (2010) 138:1704–13. 10.1053/j.gastro.2009.12.004 20006613

[52] Mark SD Qiao YL Dawsey SM Wu YP Katki H Gunter EW. Prospective study of serum selenium levels and incident esophageal and gastric cancers. J Natl Cancer Inst. (2000) 92:1753–63. 10.1093/jnci/92.21.1753 11058618

[53] Wei WQ Abnet CC Qiao YL Dawsey SM Dong ZW Sun XD. Prospective study of serum selenium concentrations and esophageal and gastric cardia cancer, heart disease, stroke, and total death. Am J Clin Nutr. (2004) 79:80–5. 10.1093/ajcn/79.1.80 14684401

[54] Limburg PJ Wei W Ahnen DJ Qiao Y Hawk ET Wang G. Randomized, placebo-controlled, esophageal squamous cell cancer chemoprevention trial of selenomethionine and celecoxib. Gastroenterology. (2005) 129:863–73. 10.1053/j.gastro.2005.06.024 16143126

[55] Qiao YL Dawsey SM Kamangar F Fan JH Abnet CC Sun XD. Total and cancer mortality after supplementation with vitamins and minerals: follow-up of the linxian general population nutrition intervention trial. J Natl Cancer Inst. (2009) 101:507–18. 10.1093/jnci/djp037 19318634 PMC2664089

[56] Cai X Wang C Yu W Fan W Wang S Shen N. Selenium exposure and cancer risk: an updated meta-analysis and meta-regression. Sci Rep. (2016) 6:19213. 10.1038/srep19213 26786590 PMC4726178

[57] Hong B Huang L Mao N Xiong T Li C Hu L. Association between selenium levels and oesophageal adenocarcinoma risk: evidence from a meta-analysis. Biosci Rep. (2016) 36:4. 10.1042/BSR20160131 27190131 PMC4937171

[58] Park Y Leitzmann MF Subar AF Hollenbeck A Schatzkin A. Dairy food, calcium, and risk of cancer in the NIH-AARP diet and health study. Arch Intern Med. (2009) 169:391–401. 10.1001/archinternmed.2008.578 19237724 PMC2796799

[59] Lipkin M. Application of intermediate biomarkers to studies of cancer prevention in the gastrointestinal tract: introduction and perspective. Am J Clin Nutr. (1991) 54:188S−92S. 10.1093/ajcn/54.1.188S 2053560

[60] Yang CY Chiu HF Tsai SS Wu TN Chang CC. Magnesium and calcium in drinking water and the risk of death from esophageal cancer. Magnes Res. (2002) 15:215–22. 12635875

[61] Proctor DM Suh M Mittal L Hirsch S Valdes Salgado R Bartlett C. Inhalation cancer risk assessment of hexavalent chromium based on updated mortality for Painesville chromate production workers. J Expo Sci Environ Epidemiol. (2016) 26:224–31. 10.1038/jes.2015.77 26669850 PMC4756268

[62] Chen QY Murphy A Sun H Costa M. Molecular and epigenetic mechanisms of Cr(VI)-induced carcinogenesis. Toxicol Appl Pharmacol. (2019) 377:114636. 10.1016/j.taap.2019.114636 31228494 PMC6658109

[63] Ge X He J Wang L Zhao L Wang Y Wu G. Epigenetic alterations of CXCL5 in Cr(VI)-induced carcinogenesis. Sci Total Environ. (2022) 838:155713. 10.1016/j.scitotenv.2022.155713 35660107 PMC9290188

[64] Sun G Wang Y Hu W Li C. Effects of manganese superoxide dismutase (MnSOD) expression on regulation of esophageal cancer cell growth and apoptosis in vitro and in nude mice. Tumour Biol. (2013) 34:1409–19. 10.1007/s13277-012-0622-x 23649652

[65] Schiffman SC Li Y Martin RC. The association of manganese superoxide dismutase expression in Barrett's esophageal progression with MnTBAP and curcumin oil therapy. J Surg Res. (2012) 176:535–41. 10.1016/j.jss.2011.11.1013 22316666

[66] Murphy R. An integrative approach to assessing diet-cancer relationships. Metabolites. (2020) 10:123. 10.3390/metabo10040123 32218376 PMC7241082

[67] Irimie AI Braicu C Pasca S Magdo L Gulei D Cojocneanu R. Role of key micronutrients from nutrigenetic and nutrigenomic perspectives in cancer prevention. Medicina. (2019) 55:6. 10.3390/medicina55060283 31216637 PMC6630934

